# Complete resection of an incidentally discovered posterior mediastinal ganglioneuroma in an asymptomatic child: a case report

**DOI:** 10.1093/jscr/rjaf685

**Published:** 2025-09-02

**Authors:** Ahmad Bishr Nasra, Ahmad Al-Bitar

**Affiliations:** Faculty of Medicine, Damascus University, Damascus, Syrian Arab Republic; Faculty of Medicine, Damascus University, Damascus, Syrian Arab Republic

**Keywords:** ganglioneuroma, posterior mediastinum, pediatric tumor, incidental finding, thoracotomy

## Abstract

Ganglioneuromas (GNs) are rare, benign tumors originating from sympathetic ganglia, typically discovered in symptomatic children due to compressive effects. We report an unusual case of an 8-year-old girl with a large posterior mediastinal GN, incidentally discovered on chest X-ray during a routine evaluation. Computed tomography imaging revealed a well-defined, 11 × 7 × 5 cm homogeneous mass extending from the aortic arch to the T10 vertebral level, adherent to the descending aorta and vertebrae. A left posterolateral thoracotomy was performed for complete excision without complications. Histopathology confirmed mature GN. The patient had an uneventful recovery and remains asymptomatic on follow-up. This case highlights the importance of thorough evaluation of incidental mediastinal findings and emphasizes surgical expertise in resecting large, adherent masses safely. It also underscores the potential for asymptomatic presentation even in sizable tumors, a rare occurrence in pediatric patients.

## Introduction

Ganglioneuromas (GNs) are benign, slow-growing tumors that arise from neural crest derivatives, including ganglion cells, Schwann cells, and fibrous tissue [1–3]. First described in 1870, these neoplasms are exceptionally rare, with an estimated incidence of one case per million individuals, accounting for just 0.1%–0.5% of all neurogenic tumors [1, 2]. GNs predominantly affect children: 60% of diagnoses occur before the age of 20, with a median age at presentation of ⁓7 years [4]. The incidence is higher in females than in males, with a reported ratio of 3:2 [5].

Clinically, GNs are often detected due to compressive symptoms on adjacent organs or structures, prompting surgical intervention [1, 4]. Early detection in pediatric patients typically limits tumor growth within available body-cavity space; however, very large lesions have been documented, with one of the largest measuring ⁓23 cm in diameter [6]. The posterior mediastinum is a recognized site for GN occurrence [7]. An incidentally discovered mediastinal mass in an otherwise healthy child thus poses a significant diagnostic and therapeutic challenge. We report the case of an 8-year-old girl diagnosed with a large, asymptomatic posterior mediastinal GN, which was incidentally identified on a chest X-ray. The mass's firm attachment to the descending aorta and thoracic vertebrae necessitated a complex surgical intervention for successful removal.

## Case presentation

An 8-year-old Arab girl with no relevant medical history was referred after a chest X-ray revealed a mediastinal mass. The X-ray had been performed following a minor fall at school in which she struck her side. Although she was completely asymptomatic, the imaging was done out of precaution and unexpectedly revealed a posterior mediastinal opacity. She had no respiratory distress, chronic cough, dysphagia, or neurologic complaints. Physical examination was unremarkable, with no palpable masses or deficits.

**Figure 1:**
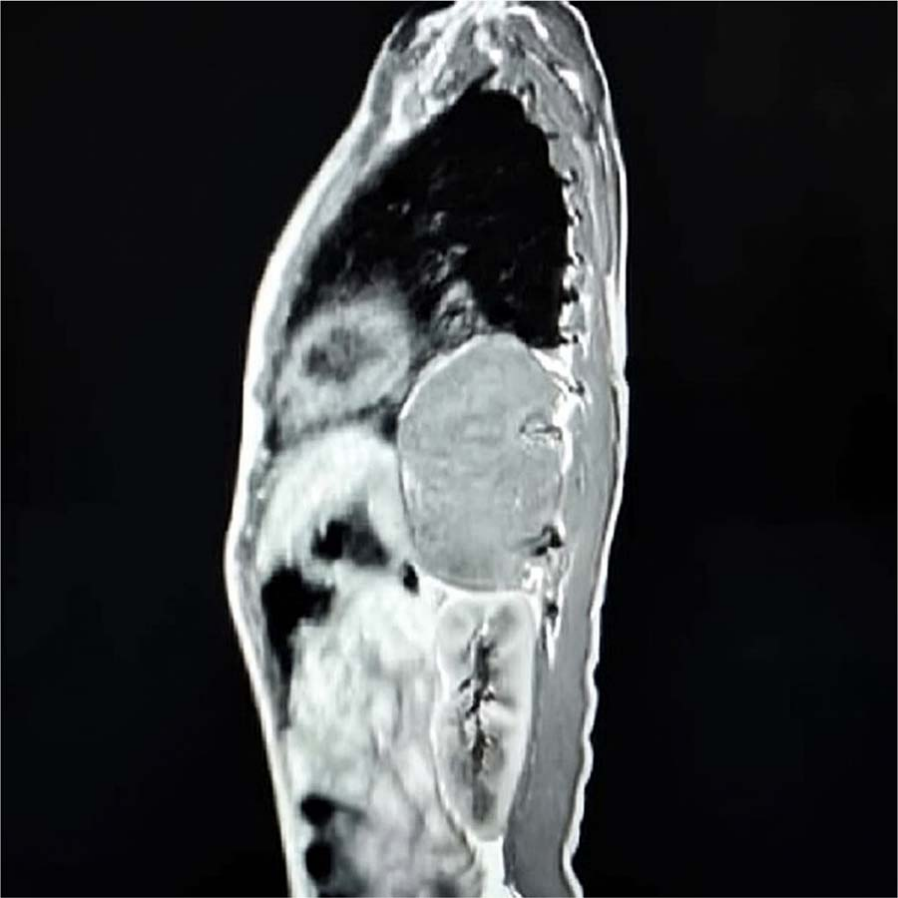
Sagittal contrast-enhanced CT scan showing a well-defined, homogeneous, soft-tissue mass (11 × 7 × 5 cm) in the left posterior mediastinum, abutting the descending aorta and thoracic vertebrae.

Contrast-enhanced computed tomography (CT) confirmed a well-circumscribed, homogeneous soft-tissue mass measuring 11 × 7 × 5 cm in the left posterior mediastinum, extending from the level of the aortic arch to the tenth thoracic vertebra and occupying much of the left pleural space (Fig. 1). The lesion was adherent to the thoracic vertebral bodies and descending aorta, but without evidence of vascular invasion or bony erosion. Differential diagnoses included neuroblastoma, GN, and—less likely—lymphoma.

**Figure 2:**
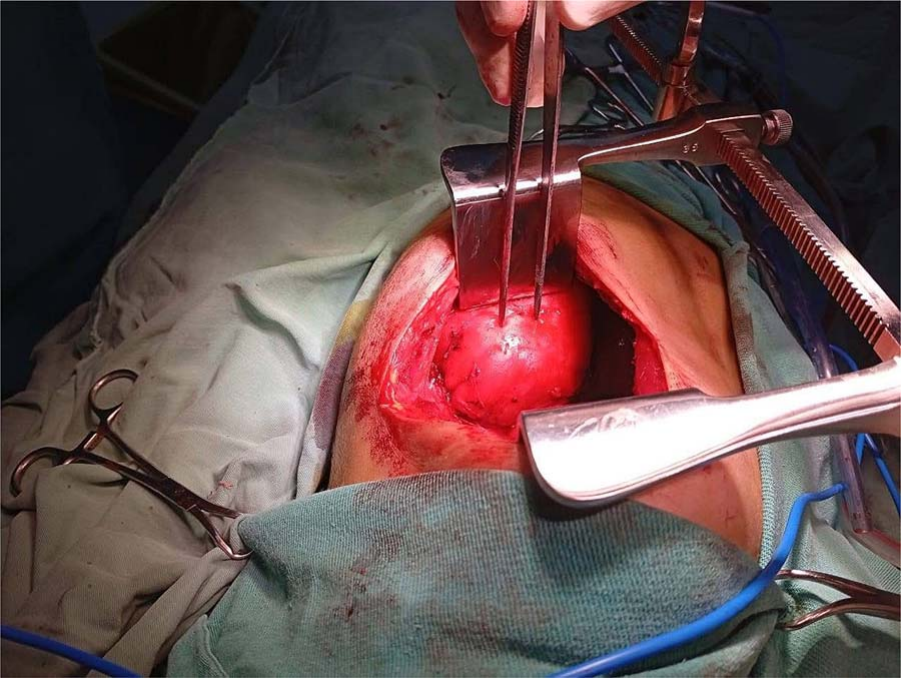
Intraoperative view during left posterolateral thoracotomy showing the encapsulated posterior mediastinal mass after exposure and mobilization.

**Figure 3:**
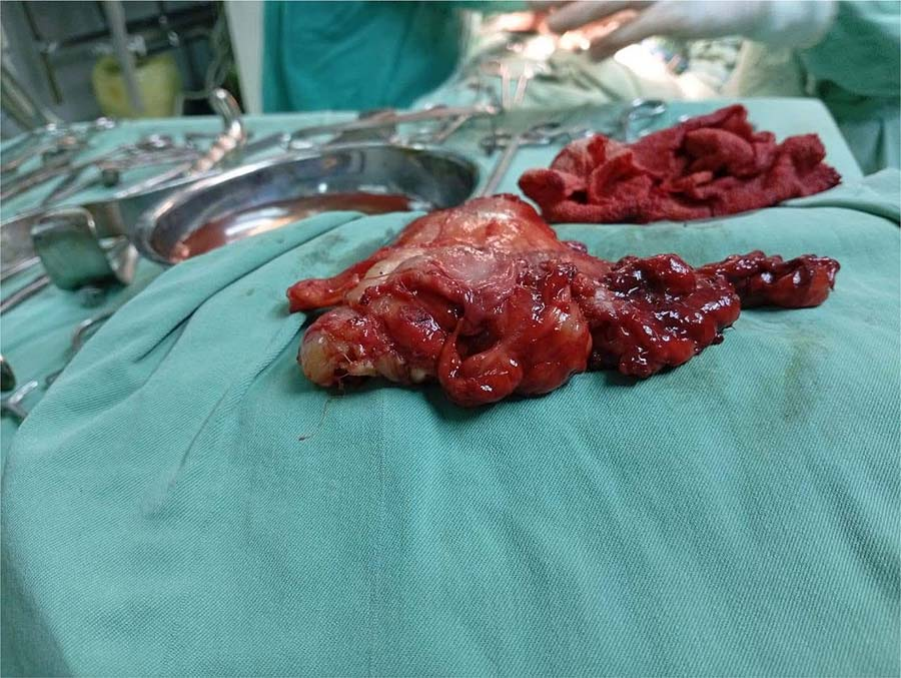
Gross intraoperative specimen of the posterior mediastinal mass following en bloc resection.

The patient underwent a left posterolateral thoracotomy. Intraoperatively, the mass was firm, encapsulated, and vascularized, with dense adhesions to the vertebrae and aorta. Meticulous dissection facilitated en bloc resection without vascular or neurologic complications (Figs 2 and 3). The postoperative course was uneventful: the chest tube was removed on postoperative day two, and she was discharged in excellent condition on day three.

Histopathology confirmed a mature GN consisting of ganglion cells, Schwann cells, and nerve fibers, with no immature or malignant elements. No adjuvant therapy was required, and the prognosis is excellent, with a very low risk of recurrence.

## Discussion

Glioneuroma is a rare, benign neurogenic tumor that originates from sympathetic ganglia and, less commonly, the adrenal medulla [8–10]. As the most differentiated form within the neuroblastic tumor spectrum, GNs exhibit minimal malignant potential. These tumors predominantly affect children and typically present as solitary lesions along the sympathetic chain, most often in the posterior mediastinum (41.5%), retroperitoneum (37.5%), or adrenal gland (21%), with rarer involvement of the cervical region, retropharyngeal space, or sella turcica [11–14]. Although GNs may harbor neuroendocrine potential, they are generally hormonally inactive; a small subset is associated with genetic syndromes such as multiple endocrine neoplasia type 2 or neurofibromatosis types 1 and 2 [3, 15].

Clinically, GNs often manifest through mass effect, causing respiratory or gastrointestinal symptoms. Rare paraneoplastic presentations include diarrhea, hypertension, virilization, and myasthenia gravis. However, asymptomatic, large tumors are uncommon: a systematic review of 364 cases reported incidental detection in only 24.5% (20.3%–39.1%) [16].

Cross-sectional imaging (CT or MRI) is the diagnostic gold standard, typically revealing well-defined, homogeneous or mildly heterogeneous hypodense masses. Definitive diagnosis requires histology to exclude ganglioneuroblastoma and paraganglioma, with benignity confirmed by the absence of mitotic figures, immature neuroblasts, or necrosis [17, 18].

Because GNs are so rare and lack distinct imaging features, many radiologists may be unfamiliar with their appearance, which can lead to diagnostic delays or misinterpretation [19].

This highlights the importance of correlating radiologic findings with histopathological analysis to ensure an accurate diagnosis and avoid unnecessary interventions [19].

Standard chest X-rays often miss smaller lesions due to their limited sensitivity and tend to detect masses only when they have grown large enough to create visible changes [20].

When visible, neurogenic tumors on radiographs typically present as smooth, well-defined paraspinal opacities that may obscure the paraspinal lines or cause scalloping of adjacent bones [21].

CT, on the other hand, offers clearer visualization of the tumor’s size, location, and relationship to surrounding structures. These lesions often appear as sharply marginated soft-tissue masses, sometimes containing calcifications, fatty elements, or areas of necrosis [21].

Magnetic resonance imaging (MRI) is particularly valuable in assessing possible intraspinal extension and evaluating the tumor’s proximity to neural foramina [21].

Complete surgical excision is the standard treatment. Surgical planning must account for tumor size, location, and relationship to vital structures; dense adhesions to vessels or vertebrae increase procedural complexity and risk, necessitating precise preoperative assessment and meticulous intraoperative technique [22–25].

Although our patient’s surgery was completed without complications, it’s important to note that resection of GNs, particularly in the posterior mediastinum, is not without risks.

One study reported a 25.9% perioperative complication rate among pediatric patients undergoing these procedures, with common issues including pulmonary infections, respiratory failure, and extended need for ventilatory support [24].

In more complex cases, intraoperative challenges may involve significant blood loss, injury to major vessels, or even the need for cardiopulmonary bypass when tumors are closely associated with critical structures [22].

Postoperative complications can include chylothorax, pleural effusions, and, less commonly, neurological deficits such as Horner’s or Harlequin syndrome [23].

For these reasons, careful preoperative imaging, collaborative multidisciplinary planning, and the involvement of experienced surgical teams are essential to minimize risks and ensure optimal outcomes.

Our patient’s 11 × 7 × 5 cm posterior mediastinal GN was incidentally discovered in an asymptomatic child—an exceptionally rare scenario given the lesion’s size. The mass’s firm adherence to the descending aorta and thoracic vertebrae required a one-stage left posterolateral thoracotomy. The technically demanding dissection achieved en bloc removal without complications, highlighting the importance of careful planning and surgical expertise. Given the benign nature of GN and the completeness of resection, long-term imaging surveillance is recommended to detect any recurrence early while avoiding needless interventions.

## Conclusion

This case illustrates the rare incidental discovery of a large posterior mediastinal GN in an asymptomatic child. Complete surgical resection was achieved despite the mass’s adherence to vital structures, demonstrating the importance of meticulous operative planning and execution. The excellent outcome supports surgery as the definitive treatment and highlights the need for follow-up to monitor for recurrence.

## Data Availability

The manuscript contained all data related to this case report.
